# Petrographic and Physical-Mechanical Investigation of Natural Aggregates for Concrete Mixtures

**DOI:** 10.3390/ma14195763

**Published:** 2021-10-02

**Authors:** Chiara Telloli, Alessandra Aprile, Elena Marrocchino

**Affiliations:** 1 ENEA, Italian National Agency for New Technologies, Energy and Sustainable Economic Development, Fusion and Technology for Nuclear Safety and Security Department, Nuclear Safety, Security and Sustainability Division—Via Martiri di Monte Sole 4, 40129 Bologna, Italy; chiara.telloli@enea.it; 2 Department of Engineering, University of Ferrara—Via Saragat 1, 44122 Ferrara, Italy; prllsn@unife.it; 3 Department of Chemistry, Pharmaceutical and Agricultural Sciences, University of Ferrara—Via L. Borsari 46, 44121 Ferrara, Italy

**Keywords:** concrete, aggregates, petrography, physical-mechanical characterization

## Abstract

The availability of different lithology with which concrete can be packaged could create substantial questions on the differences that they can provide to the same mixture. Different kinds of aggregates were analyzed individually to investigate their main characteristics, which allowed us to package five types of concrete mixtures. These five mixtures were compared to each other through compressive strength values. Furthermore, it was considered microscopically what possible differences could exist between these different mixtures, for example, differences in the cement/aggregate reaction. The chemical characterization of the aggregates, used as the skeleton of the cement mixes, was proposed as an important investigative phase in order to better understand the differences in the geotechnical and physical-mechanical characteristics and to verify the presence of any harmful phases for the durability of the concrete.

## 1. Introduction

The development of society has led to a continuous improvement of the construction techniques to create voluminous buildings [1,2]. Concrete is certainly one of the most important building materials in the modern era [3,4].

Since ancient times, the use of binders and similar materials characterized the history of man’s settlements, but only with Romans concrete was used as a modern connotation [5,6]. In fact, Romans used stone scraps called *caementum* mixed with lime and a *pulvis puteolana* (pozzolanic powder) which constituted the binder of the mixture [7,8].

Nowadays, concrete indicates a conglomerate formed by cement, aggregates (gravel or crushed stone, sand) and water which, hardening, gives rise to a real artificial stone [9]. In modern concrete, the addition of artificial and natural fibers or additives to improve its mechanical and workability characteristics is also fundamental [10,11].

The apparently simple composition of the concrete underestimates the problems of degradation that can arise in the short and long term due to the presence of macro and micro pollutants or to reactivity with the aggregates [12,13]. After about a century of using concrete in the engineering field, there is a need to know every single detail of the fundamental constituent, especially chemical and petrographic, in order to avoid the risk of creating poor mixtures, as, after a more or less short period from installation, they may lose some of their physical-mechanical and/or structural-compositional properties [14].

The Council Directive 89/106/EEC declares that construction products must possess characteristics that impose important requirements of mechanical strength and stability, safety in case of fire, energy saving, protection against noise, hygiene and safety in use [15]. On May 2004, the CE marking for aggregates came into force, or the certification of the performance characteristics of each product. The fundamental characteristics that the aggregates must have are: size and shape of the particles; resistance to fragmentation/crushing; resistance to sanding/abrasion; chemical composition; presence or absence of dangerous substances; frost durability; and durability against alkali–silica reaction [16].

In addition, the UNI EN 12620:2013 deals with the correct qualitative characterization of the aggregates that must be used in the packaging of concrete [17].

For all these reasons, a correct assessment of the medium- and long-term effects is necessary to verify the suitability of the materials both at the delivery of the work and after periods of exposure to the mechanical stresses imposed by anthropogenic activities [18] and to the chemical-physical variations that may be imposed on the environment [19].

This study aimed to characterize the lithic component of the concrete with specific laboratory tests aimed at highlighting its strengths and defects in the best possible way. Furthermore, we wanted to petrographically and mechanically compare mixtures of concrete made with aggregates of different types and different genesis: e.g., natural conglomerates with a high form factor with a smooth surface, originated from quarries in different regions; and artificial breccia obtained by crushing natural conglomerates and characterized by irregular morphology and high surface roughness.

The aggregates used in the construction field come from the processing of natural deposits geologically called conglomerates, consolidated sedimentary deposits consisting of a preponderant content of more or less rounded pebbles and gravels [20,21]. The alluvial conglomerates available for the Italian aggregate market have a strong lithological diversity as most are made up of well-rounded river pebbles or conglomerates whose composition substantially reflects the type of outcrops in the catchment basin [22]. The aggregates used for this research work came from quarries in different geographical areas of northern Italy: Nervesa della Battaglia (Treviso), Santhià (Vercelli) and Segonzano (Trento), which, respectively, produce concrete with aggregates from calcareous, metamorphic and artificial aggregates of porphyry. The studied conglomerates respond to mixes actually existing and used in the construction sector and in which the inert aggregates are selected to provide a good skeleton to the mixture, thus trying to achieve the best possible mechanical characteristics with the imposed water/cement ratio (w/c).

The suitability of the different types of aggregates was determined using the main laboratory tests, physical-mechanical and thermal, which the UNI EN standard normally requires from producers of aggregates [23,24,25,26,27]. For the characterization of each lithological group, petrographic investigations that are also required by the UNI EN standards were carried out [28]. Finally, thanks to the use of a stereomicroscope, the possible relationships that aggregates of different shapes (rounded or artificially crushed) and cement paste are able to offer were observed. Finally, physical-mechanical characteristics of the mixtures made with aggregates deriving from conglomerates of different origins were determined through mechanical resistance tests.

## 2. Materials and Methods

### 2.1. Samples Description

Three different types of aggregates were compared: (a) conglomerate of carbonate sedimentary rocks from Nervesa della Battaglia (Treviso, Italy); (b) conglomerate of silicate and carbonate metamorphic rocks from Santhià (Vercelli, Italy); and (c) artificial porphyry aggregates from waste processing in the extraction of slabs and blocks for ornaments from Segonzano (Trento, Italy).

**Figure 1:**
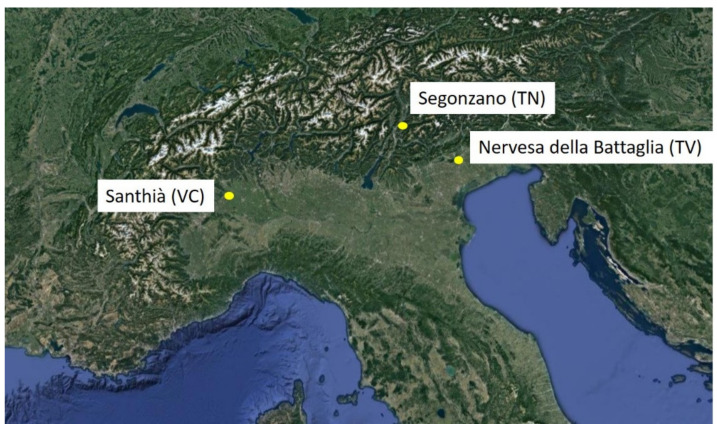
Map of the three different sampling sites colored in yellow, located in the north of Italy.

Figure 1 shows the three different sampling sites of the analyzed material, all located in the northern part of Italy.

**Figure 2:**
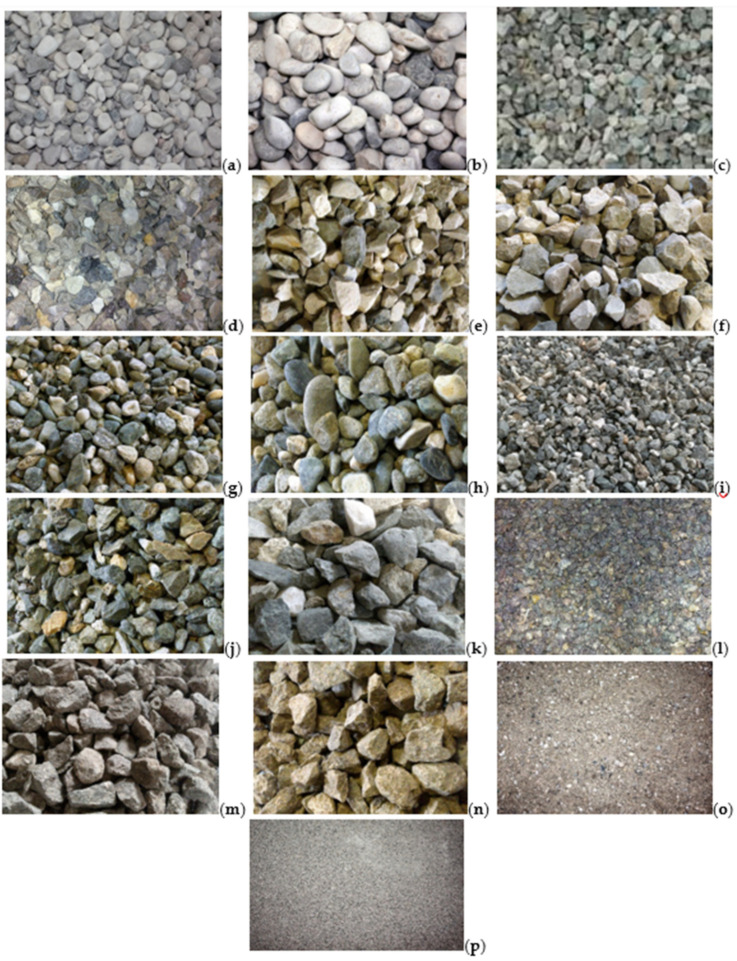
Photo imaging of the samples analyzed: (**a**) calcareous gravel 6/16; (**b**) calcareous gravel 16/32; (**c**) calcareous crushed stone 4/8; (**d**) calcareous crushed stone 8/12; (**e**) calcareous crushed stone 12/20; (**f**) calcareous crushed stone 20/28; (**g**) metamorphic gravel 5/16; (**h**) metamorphic gravel 15/30; (**i**) metamorphic crushed stone 5/9; (**j**) metamorphic crushed stone 9/16; (**k**) metamorphic crushed stone 16/25; (**l**) porphyry 4/8; (**m**) porphyry 8/20; (**n**) porphyry 16/31; (**o**) calcareous sand 0/6; (**p**) calcareous sand 0/1.

Figure 2 shows the photos of the aggregates used. The calcareous aggregates came from the processing and selection of Quaternary alluvial conglomerate gravels deposited in the Veneto–Friuli plain by fluvioglacial landform currents originating from the melting of the Piave Glacier (Würm) [29]. Samples of natural and inert gravels coming from the crushing of the calcareous gravels were selected in different sizes: calcareous gravel 6/16 (Figure 2a); calcareous gravel 16/32 (Figure 2b); calcareous crushed stone 4/8 (Figure 2c); calcareous crushed stone 8/12 (Figure 2d); calcareous crushed stone 12/20 (Figure 2e); and calcareous crushed stone 20/28 (Figure 2f).

The metamorphic aggregates, on the other hand, came from the selection of alluvial conglomerate gravels of the Vercelli plain originating by the fluvioglacial activity between the Riss and Würm glaciations (moraine amphitheater of Ivrea) [30]. Samples of natural and inert gravels coming from the crushing of metamorphic gravels were selected in the following sizes: metamorphic gravel 5/16 (Figure 2g); metamorphic gravel 15/30 (Figure 2h); metamorphic crushed stone 5/9 (Figure 2i); metamorphic crushed stone 9/16 (Figure 2j); and metamorphic crushed stone 16/25 (Figure 2k).

Finally, the porphyry aggregates were obtained from the processing of rhyolitic ignimbritic deposits of the Atesina porphyritic platform that originated following the Permian volcanic activity [31]. The crushed aggregates analyzed were selected in the following sizes: porphyry 4/8 (Figure 2l); porphyry 8/20 (Figure 2m); and porphyry 16/30 (Figure 2n).

In addition, two different calcareous sand samples were used, characterized by different sizes 0/6 and 0/1, as shown in Figure 2o,p, respectively.

For each of these three types of aggregates, concrete mixtures were made both with aggregates from the original conglomerates and with materials obtained from their crushing.

The preparation of a single type of mixture with the use of the same water/cement ratio (w/c) but with different aggregates highlights possible strengths and weaknesses of some lithologies rather than others. The description of the mixtures was better described in Section 2.3.

For the fine fraction, a medium-coarse sand with calcareous gravel and a medium-fine sand (nomenclature according to Wentworth dimensional classification [32]) were used, both collected in the mining area of Nervesa della Battaglia (Treviso, Italy).

### 2.2. Laboratory Tests

First of all, specific analyses were carried out on the individual aggregates to evaluate all their characteristics in order to then be able to establish the parameters necessary to create the concrete mixes. All the analyses were carried out at the Laboratory of the Department of Physics and Earth Sciences of the University of Ferrara (Italy).

The analyses performed on the individual aggregates were: particle size analyses [33,34,35,36,37], determination of the physical-mechanical properties [23,24,25,26], description of the thermal and degradability properties of the material and petrographic analyses [27,28].

Petrographic analysis is important to identify the presence or not of minerals responsible for the alkali–silica reaction [38,39]. It is generally associated with the presence of alkali (sodium and potassium) in the cement and of amorphous or poorly crystalline silica in some aggregates. Amorphous silica is present where minerals, such as opal or microcrystalline quartz, are observed and also by the presence of volcanic glass and siliceous fragments of fossils, such as remains of radiolarians, diatoms and spicules of sponges [40]. These constituents can interact with the alkaline elements of the cement to form a sort of frozen alkaline silicate capable of swelling in a humid environment [41]. The optical stereomicroscope Optika SZ6745TR (Ponteranica, Bergamo, Italy) equipped with Moticam 2500 5.0 M pixel webcam was used for reflected light observation on thin section. The Motic Images Plus 2.0 ML software (Motic Instruments Inc., Richmond, Canada) was used to analyze the observation obtained [42]. Observation on thin sections was conducted using transmitted light polarized microscopy (BX51 Olympus, Tokyo, Japan).

### 2.3. Mixture Creation

The analyses of the single aggregates made it possible to identify the characteristics of the analyzed material. Thanks to this, it was possible to obtain five mixtures on which the compressive strength tests and petrographic analyses were carried out.

To prepare the concrete, the compressive strength class had to first be chosen from which the minimum w/c ratio to use was deduced. Then the optimal particle size curve was determined, obtained using a predetermined theoretical curve as a reference (Bolomey’s curve) [43,44].

Five mixtures were obtained using the constant parameters set out above and the formulation of an ordinary type of concrete: XC2 mixture with resistance class C25/30 [45], minimum w/c ratio of 0.60 and minimum cement content of 300 Kg/m^3^ (values indicated in the UNI EN 11104:2016 [46]). Another important step was to calculate the liters of water necessary to maintain the desired w/c ratio, knowing the minimum quantity of cement provided by the UNI EN 206-1:2006 [47] (or UNI EN 11104:2016 for the application in Italy of the UNI EN 206-1:2006). The quantities by weight of the aggregates were determined starting from the respective volumetric quantities deducted from the percentages of each fraction in the granulometric curve, and, knowing the bulk density of each fraction used, the quantity by weight per unit volume was obtained.

In compliance with the formulation of an ordinary concrete such as those packaged in concrete mixing plants, it was decided to add a plasticizing additive equal to an amount of 0.8% on the weight of the cement in order to obtain an increase in the consistency class with relative improvement in the workability characteristics.

The same quantities of the two different types of sand samples were used for all the mixtures to ensure the same type of fine-fraction constant, thus varying only the granular material. This arrangement was necessary to establish an initial starting point for the correct comparison of the mechanical results deduced from the subsequent simple compression test.

Two different quantities of cement and water were used for the mixtures with crushed aggregates and with rounded aggregates of the gravel. The quantity of water was increased or decreased by 10 kg/m^3^, respectively, for crushed aggregate or rounded gravel aggregate. This was because the flow between the crushed aggregates was less than that between the rounded aggregates due to their different geometric shape: multi-sided and polyhedral for the former and smoother for the latter. The increase in friction between the polyhedral aggregates would lead to a decrease in the degree of sliding on each other and therefore to a consequent decrease in workability and in the relative consistency of the slump classification. These different quantities of water were therefore related to two different quantities of cement in order to obtain a constant w/c ratio for all set at 0.58.

Table 1, Table 2, Table 3, Table 4 and Table 5 summarize the dosages by weight of the various components (aggregates, water, cement and additive) referred to a volume of 1 m^3^ of concrete and equivalent to 1000 L.

To mix the components, a laboratory concrete mixer was used which made it possible to obtain mixtures of 50 L each, a quantity necessary for the formation of a fair number of cubes for each type of mixture.

For each mixture obtained, the workability measurement was immediately carried out through the *Abrams cone lowering test* (UNI EN 12350-2:2019), which consists of the measurement of the fall of a fresh concrete cone suitably formed inside a conical mold with standardized dimensions [48]. The concrete cone was settled into the mold as much as plastic, and therefore the mixture was workable. The measure determined from the highest point of the collapsed mixture to the maximum possible height of the cone, given by the metal mold, will fall within one of the concrete slump classes (S) [47]. A consistency class of S4 was obtained for all the mixtures, characterized by a lowering of the cone between 160 and 210 mm, and which therefore confers a fair fluidity particularly useful in conditions of strong reinforcement of the work. This consistency was mainly attributable to the use of the fluidifying additive, which allowed for an increase in the workability of the mixture in place of the reduced introduction of water into the mixture. This aspect had a positive effect on the quantity of cement to be used; in fact, to keep the w/c ratio unchanged, the decrease in water led to a corresponding decrease in cement, with positive implications from a practical (less heat of hydration) and economic point of view.

## 3. Results

First, the results obtained for the single aggregates are shown, then for the created mixtures.

### 3.1. Characterization of the Single-Aggregate Samples

#### 3.1.1. Particle Size Analyses

Various properties of the concrete depend on the particle size distribution of the aggregate and in particular its workability, that is, the ability to be mixed, installed and processed. A mixture of only a coarse aggregate would be difficult to work because the lack of fine particles inhibits the flow of an inert material on the other; in addition, a mixture without internal adhesion and therefore inconsistent and easily disintegrated would be created. On the other hand, a mixture with abundant fine particles prefers a lot of water to be able to overcome the internal resistance due to the increase in the surface of the particles. Finally, an optimal particle size distribution resulting from the correct calibration of percentages of the different sizes available is the best way to achieve the desired workability and resistance characteristics [49].

**Figure 3:**
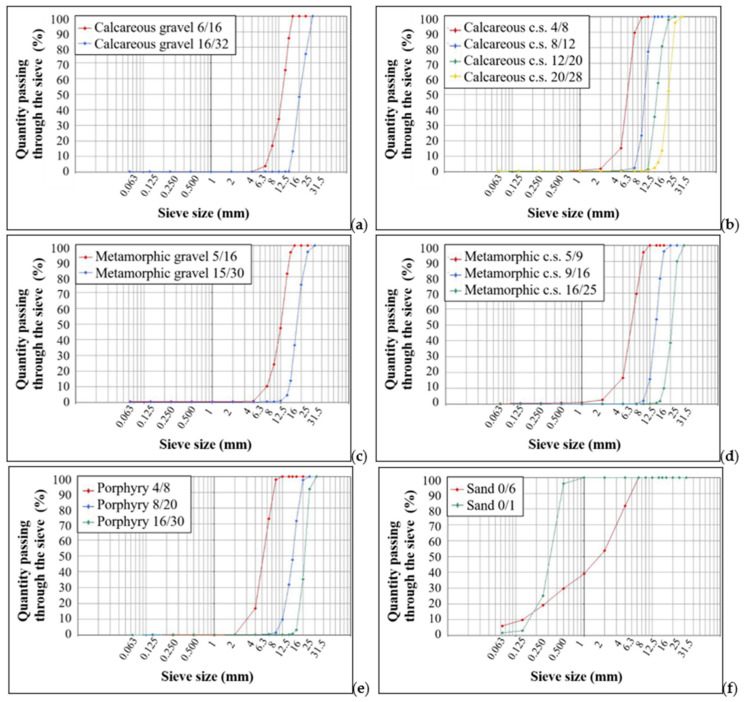
Grain size distributions of the samples analyzed, following the Bolomey’s curve: (**a**) calcareous gravel samples; (**b**) calcareous crushed stone samples (c.s. in the graph means crushed stone); (**c**) metamorphic gravel samples; (**d**) metamorphic crushed stone samples (c.s. in the graph means crushed stone); (**e**) porphyry samples; (**f**) sand samples.

Table 6 and Table 7 show the particle size dimensions of the aggregates analyzed with the relative grain size distributions (Figure 3).

#### 3.1.2. Physical-Mechanical Properties

Table 8 shows that the aggregates have low shape index (SI) and flattening coefficient (FC) values. This could be related to the low number of elongated individual particles that characterize the samples. From the comparison, however, it could be noted that the metamorphic aggregates have slightly higher values than the calcareous and porphyritic aggregates, probably due to their lithological nature. In fact, they consist of different types of metamorphic rocks including shales, which have a flatness that generally controls the direction of erosion of the clasts or of any artificial breakage of the aggregate itself [22]. This therefore confirms that the shape of the inert material depends on the type of source rock from which it comes: uniform and isotropic rocks such as calcareous materials generally produce aggregates tending to sphericity, while anisotropic rocks generally produce aggregates of different shapes, discoidal in the case of densely stratified source rocks [50,51].

The crushed-stone aggregates of fine grain size are in all three cases characterized by higher values than the respective coarser fractions, and this could be explained by the crushing process, which in the smallest elements tends to form splinters that are difficult to work due to their minute size [52,53].

From the analysis of the results of the sand samples, however, the presence of a low and non-reactive percentage of very fine materials was observed (ES value) [37]. Based on the quantity in sand, the quantity of clay particles presents in the sample, which can create concrete damage, was estimated. The ES value was obtained between the different separation between coarse and sandy material deposited at the bottom during the test and the very fine material in suspension. A material that has high suspension values has low ES values, a sign that the material contains a good percentage of very fine particles, mostly possibly clayey because they are able to flocculate within the appropriate solution [54]. Table 9 shows that the ES value for sand 0/1 is better than that of the other sand sample. These values are supported by the particle size analysis in which the values related to the 0.063 mm sieve passages are very low (Table 6). The presence of fine materials is important in the construction of the optimal granulometric curve of the concrete mixture because it helps to bind the coarse skeleton and fills the intra-clast voids.

The MB value was determined according to the operating procedure of the methylene blue test [36] for the two different sand samples. As a proportion of the size of the particles, the amount of methylene blue solution required increased, and thus the MB value increased for sand 0/6 with respect to sand 0/1 (Table 9). This means that sand 0/6 contains more clay with respect to sand 0/1 and it is more harmful to concrete [55].

Knowing the bulk density and the water absorption value is important to better create the mixtures. High water absorption generally requires more water in the mixture to make up for that which is lost within the porosity of the grains and therefore not directly used by the cement during the hydration reaction [56,57]. This is usually valid in theoretical conditions of perfectly dry aggregates. In reality, normally the aggregates used for concrete are more or less wet depending on the atmospheric conditions. It is therefore necessary to consider the presence of two types of water: the one that occupies the porosity of the aggregate and the superficial one that causes the aggregate to appear wet. The determination of the theoretical amount of water to be added to the mixture will be depleted of the amount of surface water that the aggregate has, hence the importance of a correct calculation of the absorption value of each aggregate, as it can certainly affect the predetermined w/c ratio [58,59].

Table 10 reports the bulk density and water absorption value (WA) calculated for each sample analyzed.

#### 3.1.3. Thermal and Degradability Properties

The Los Angeles values [25], wear-resistance coefficients for Micro-Deval [23], polishing [26] and resistance to freezing/thawing [27] for each type of aggregate were analyzed. The samples were obtained from a mixture of all the particle size fractions available for each lithology.

From the observation of the Los Angeles coefficient (LA) reported in Table 11, typical values of good quality of aggregates are observed [60]. There is also a slight difference between calcareous aggregates and metamorphic aggregates, with a slightly higher LA coefficient in the calcareous aggregates. However, it should be noted that this LA coefficient is made possible by the presence of microcrystalline limestones, dolomites and some magmatic components such as granite and porphyry present in small percentages. Furthermore, a low LA coefficient of porphyry is observed, as expected for a resistant rock if free from alteration. Even the metamorphic aggregates have a good LA coefficient, probably helped by the lithological nature of the aggregate which is free from stratified rocks such as phyllites, which if crushed could create many small individuals and therefore pass through the 1.6 mm reference sieve.

In Table 12, the wear-resistance coefficients for Micro-Deval (MDE), as abrasion loss in the presence of water, are provided. Both for calcareous and metamorphic aggregates, the lowest value in percentage is shown for gravels [61]. Calcareous gravel could have a lower value due to its greater sphericity than the aggregates that constitute it, a finding supported also by the SI and FC shape values (Table 8), which are lower than those of metamorphic gravels.

Table 13 reports the polishing value (PL) for the different samples analyzed. Porphyry aggregates show the highest value with respect to the other aggregates, probably due to the high presence of micro and macro roughness [62,63].

This test is essential for artifacts that will undergo wear and tear lasting over time such as road surfaces, but it was deliberately considered in this work as a valid support to the proposed studies.

Table 14 shows the resistance-to-freezing/thawing values obtained for the samples analyzed. There are no obvious differences between the different sample types of aggregates.

#### 3.1.4. Petrographic Analyses

**Figure 4:**
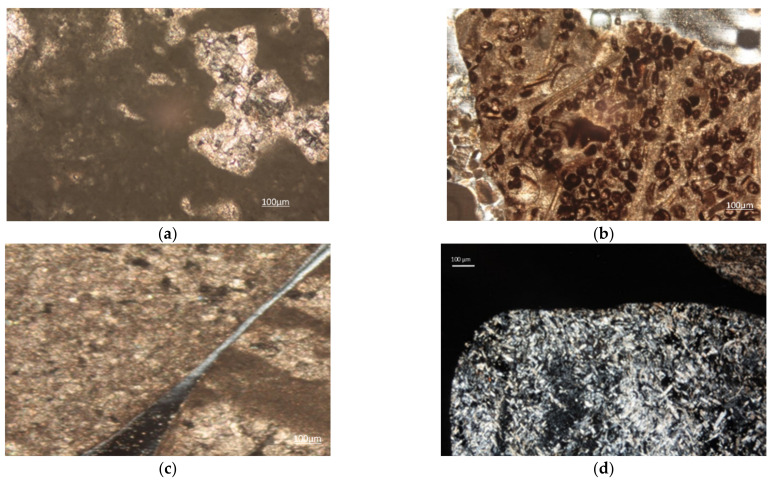

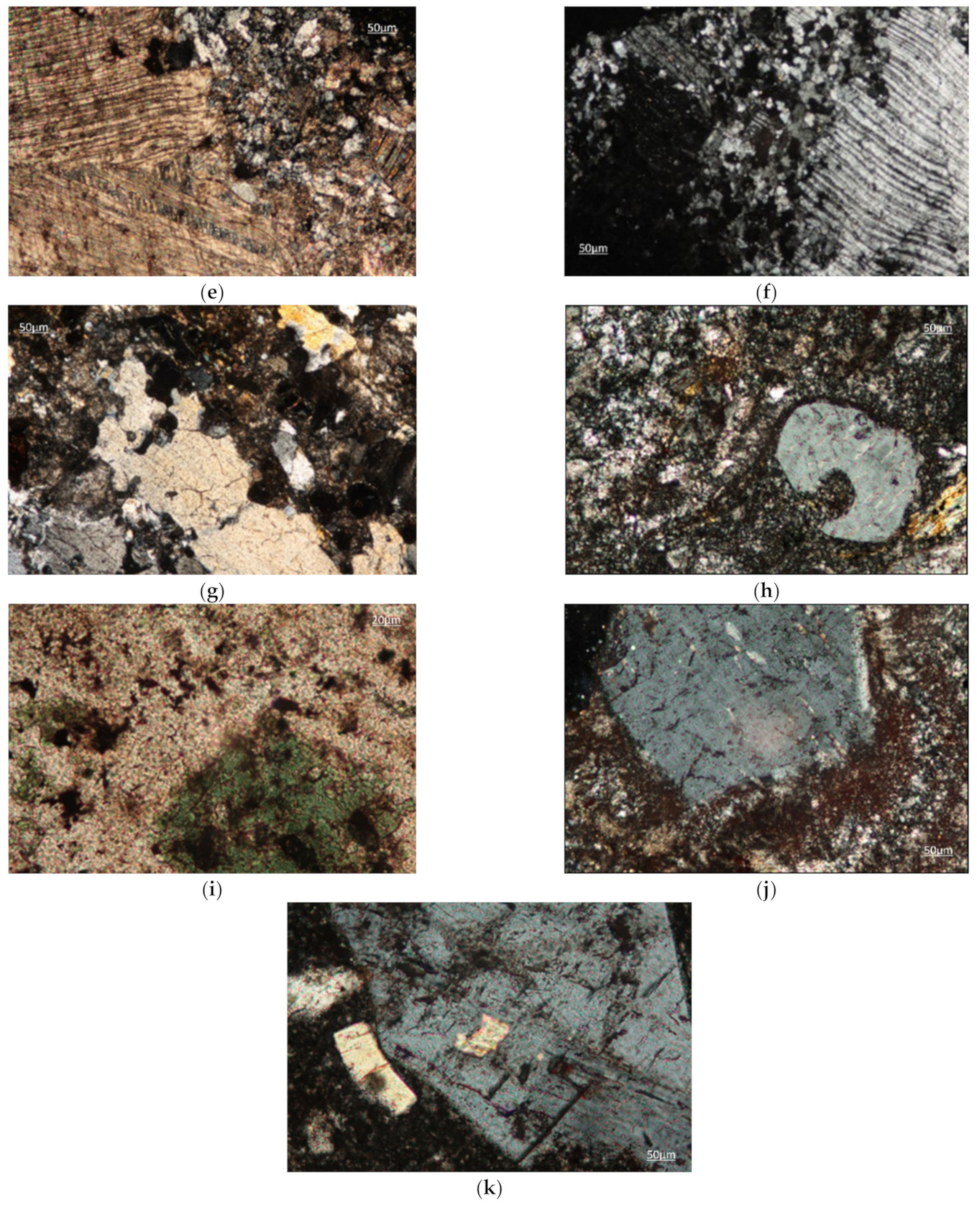
Photo imaging obtained by an optical transmitted light microscope at crossed nicols at 10× magnification: (**a**) micrite limestone with sparitic calcite; (**b**) limestone with bioclasts of shells and fragments of shells and pellets; (**c**) crystalline calcite; (**d**) serpentinite. At 10× magnification: (**e**) impure marble; (**f**) mylonites (plagioclase with undulated twinning from tectonic stress and fragments); (**g**) mylonites (quartz and k-feldspar with fragments); (**h**) porphyry that contains phenocrysts with resorption loops; (**i**) chlorite (50× magnification); (**j**) microcrystalline alteration products around feldspar phenocrysts (possible oxides); (**k**) altered and fractured potassium feldspar.

Calcareous aggregates. The predominant carbonate lithology of these aggregates is confirmed, and it reflects the supply basin of the Treviso epiclastic conglomerates from which these aggregates came. In fact, clasts of carbonate sedimentary rocks of marine origin predominate [29]. There are many fragments of micrite limestones, characterized by crystals of microcrystalline calcite and which provide a powdery appearance with beige color to both parallel and crossed nicols. However, internal recrystallizations are also observed, almost certainly diagenetic, in which larger-sized spathic calcite crystals are concentrated (Figure 4a). There are also abundant clasts of organogenic limestones in which it is possible to recognize different types of allochemical constituents. In fact, there are oolith limestones, in which the classic radiated internal structures can be recognized, but above all limestone pellets and with intra-clasts such as shells and fragments of lamellar-looking shells (Figure 4b). Numerous clasts are also observed consisting of calcite with clearly visible crystals, of possible sparitic origin or simply diagenetized or dolomitized limestone (Figure 4c). Finally, extra-basin clasts are observed as fragments of volcanic rock, present in very low percentages below 5%.

Metamorphic aggregates. From the analysis of both gravels and inert aggregates produced by the artificial crushing of the gravels, the clear silicate composition can be seen, above all attributable to paragenesis of clear metamorphic development but with the subordinate presence of intrusive magmatic paragenesis. Also in this case, the analyses support the origin of the alluvial conglomerate and the possible supply basin from which the aggregate samples came [30]. The observable paragenic associations suggest heterogeneity in the metamorphic degree and in the lithotypes of mother rock present. Metamorphic individuals with initial ultrafemic and femic protolith such as serpentinites and metagabbra are also observed. A distinctive feature for serpentinites is the thickly lamellar structure with serpentine crystals with low interference colors (Figure 4d). It is also observed the presence of metamorphic lithic elements derived from sedimentary protoliths such as impure marbles (Figure 4e), quartzites and micascists. Finally, there are gneiss, with quartz, altered potassium feldspar, biotites and chlorites, in some cases with garnet. Nevertheless, the clear abundance of metamorphic clasts, intrusive magmatic components such as tonalites, with plagioclase, altered k-feldspar and amphiboles are also observed. Finally, clasts are observed with quartz and feldspar crystals associated with mineral fragments of minute size, possibly derived from cataclastic rocks such as mylonites (Figure 4f,g).

Porphyry aggregates. The porphyritic aggregates of effusive rock of the Atesina porphyritic platform are made up of phenocrysts immersed in a microcrystalline groundmass [31]. Abundant quartz and potassium feldspar are observed, in some cases with resorption loops (Figure 4h). Neoformation minerals are present as clearly visible crystals of chlorites (Figure 4i) and possible oxides (reddish color with crossed nicols) in the microcrystalline groundmass (Figure 4j). Even the k-feldspar minerals are in some cases altered internally and densely fractured (Figure 4k).

### 3.2. Characterization of Single-Aggregate Samples

As noted in the previous section, between the three lithologies chosen, there are differences in the physical-mechanical characteristics as well as the petrographic ones. In addition, for the same lithology, a concrete mixture can vary if rounded aggregates of gravel or polyhedral aggregates of stone are used. The highlighting of these different characteristics can be tackled by considering the packaging of standard mixtures with many w/c ratios and particle size distributions but varying in each of them the lithology and the geometric shape of the coarse fraction.

#### 3.2.1. Particle Size Analyses

**Figure 5:**
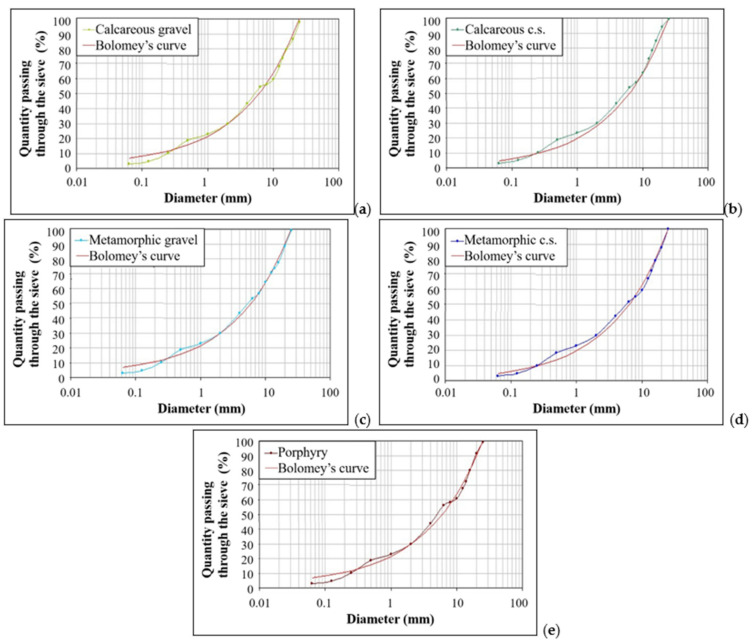
Grain size distributions of the five mixture samples analyzed following the Bolomey’s curve: (**a**) aggregate mixture with calcareous gravel (Table 1); (**b**) aggregate mixture with calcareous crushed stone (Table 2) (c.s. in the graph means crushed stone); (**c**) aggregate mixture with metamorphic gravel (Table 3); (**d**) aggregate mixture with metamorphic crushed stone (Table 4) (c.s. in the graph means crushed stone); (**e**) aggregate mixture with porphyry (Table 5).

Figure 5 shows the optimal grain size curve resulting from the mixtures created. It was decided to use the theoretical Bolomey’s curve that allows one to determine the ratio between cement and water (c/w) [64,65]. It is possible to create a workable mixture, but surely, together with the use of fluidifying additives and indirectly with the water content of the w/c ratio, it contributes to the achievement of the required consistency class [47].

For each type of mixture, the grain size distribution is similar to the Bolomey’s curve (Figure 5) and similar to each other.

#### 3.2.2. Physical-Mechanical Properties

For each mixture, the bulk density was determined through a measurement of the weight of a fresh concrete sample suitably packaged in special molds [66]. The results provide a further consideration of the quality of the concrete mixture packaging process (Table 15). Slump and bulk density are directly proportional: high value for calcareous and metamorphic mixtures and lower value of porphyry mixture both for the slump value (expressed in mm) and for the bulk density (expressed in kg/m^3^). The bulk density value obtained for all the mixtures is generally higher than that in other research works [67,68].

#### 3.2.3. Thermal and Degradability Properties

The fresh concrete samples, in the form of cubes, were subsequently matured in immersion in water at 20 °C for 28 days as required by the UNI EN 12390-3:2019 [69] and subsequently subjected to breaking under simple compression. The cubic concrete samples with a side of 150 mm were broken by means of a hydraulic press with a load advance gradient set according to the standard at 0.5 N/mm^2^. Ten cubes were packaged for each concrete mixture in order to obtain a number of data that would allow obtaining a correct representative average.

Table 16 shows the compressive strength values of each mixture. As can be seen, all the breaking values exceeded the minimum resistance limit of 30 N/mm^2^ imposed by the UNI EN 11104:2016 [46]. Furthermore, a substantial growth trend is observed, passing from calcareous to metamorphic and porphyritic lithology (see the average Rcm_28_ in Table 16). It is also important to note that, for each single aggregate mixture, the Rcm_28_ values remain more or less constant for all ten concrete cubes. In this case, the values are similar to those in other scientific works [70].

#### 3.2.4. Macroscopic Observation of the Mixture Obtained

The compressive strength test was followed by macroscopic observation of the concrete samples. These samples were obtained by prolonging the crushing phase induced by the press and which therefore allowed the analyzed cubes to be partially crushed. Some representative elements were extrapolated for each packaged mixture, and the main macroscopically observable characteristics were compared.

**Figure 6:**
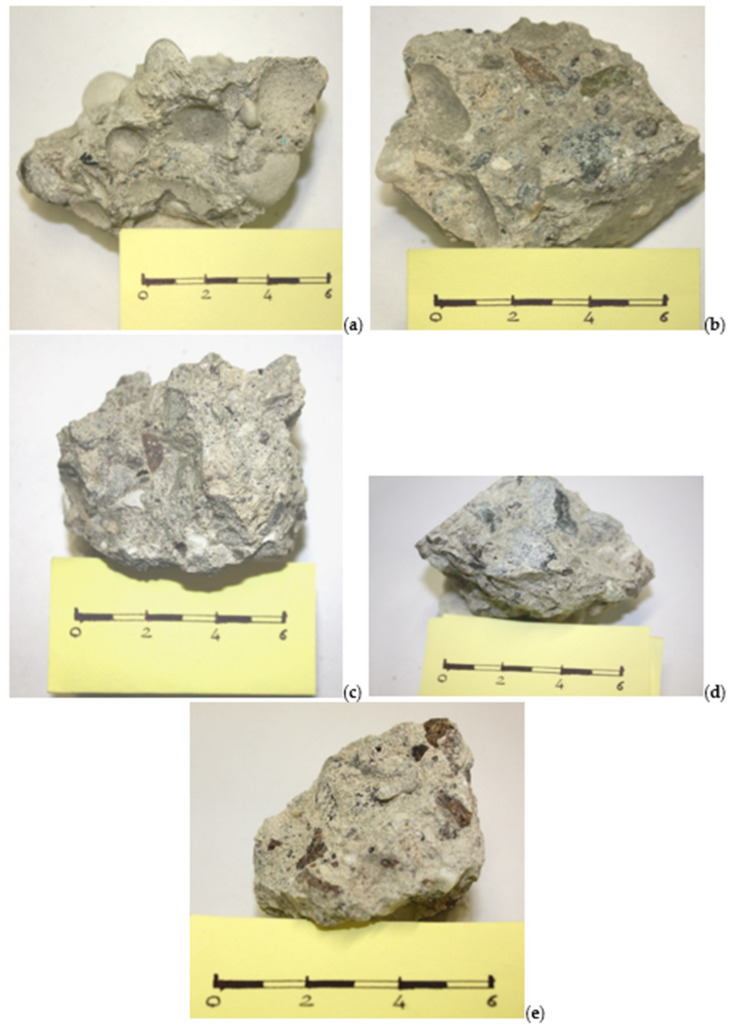
Photo imaging of the concrete mixture analyzed: (**a**) round fragment of calcareous mixture; (**b**) round fragment of metamorphic mixture; (**c**) crushed fragment of calcareous mixture; (**d**) crushed fragment of metamorphic mixture; (**e**) crushed fragment of porphyry mixture.

From the first analysis, a substantial difference is observed between the fracturing that occurred on cubes with round aggregates compared to that on cubes with crushed aggregates. The observation of the fragments shows that the detachment between cement paste and aggregate seems to be facilitated in the fragments packaged with round aggregates (gravel). This could be supported by the presence of frequent concave impressions that represent the negative mold of the aggregate itself. It therefore appears to be a detachment facilitated by the shape of the aggregate and is observed in both lithologies with rounded aggregates (Figure 6a,b).

On the other hand, in the fragments deriving from cubes packed with crushed aggregates, no evidence of detachment-impression surfaces is observed as in those with rounded aggregates (Figure 6c–e). These samples seem to have greater compactness, and the cement paste seems to contain the aggregates best. In fact, inert materials are not observed protruding much from the surface of the observed fragments as is the case for the fragments with round aggregates. At first analysis, one might think that this aspect may also depend on the particle size distribution of the aggregate which is not perfectly equal for all five mixtures obtained, but if the final curves for the calcareous lithology are compared (Figure 5), which are almost identical, this consideration could also be disproved. The rounded aggregates could therefore control the progress of the fracturing, thus creating protrusions that are not equally noticeable in the fragments with crushed aggregates [71]. Finally, the presence of a whitish patina on the surface of the aggregates is observed in the fragments with crushed aggregates, which represents what remains of the cement paste attached to the surface of the aggregate. This, which is not observed on fragments made with calcareous aggregates both rounded and crushed, is found mostly on porphyry aggregates and to a lesser extent on crushed metamorphic ones. Furthermore, on the porphyry aggregates, a greater number of fractured elements with secondary fracturing can be observed and can be distinguished by a more vivid color typical of fresh or young fractures.

#### 3.2.5. Petrographic Analyses

The detailed analysis of the thin section allowed us to focus on the aggregate/cement paste contact edges and to recognize some distinctive features that characterize the fragments with rounded aggregates and fragments with crushed aggregates [72]. There is, in fact, a different type of contact edge depending on whether the surface of the aggregate is smooth or has imperfections due to the fracture.

**Figure 7:**
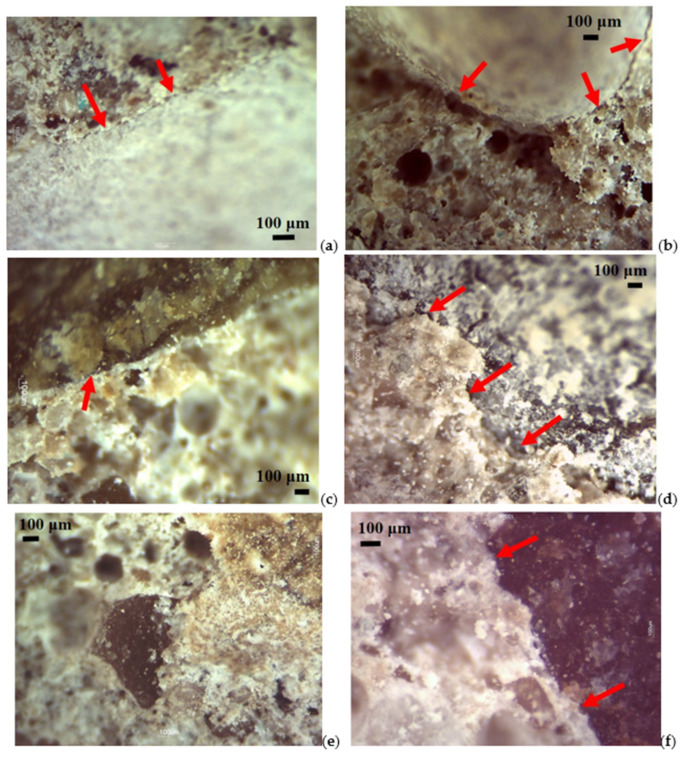
Photo imaging obtained by a stereomicroscope at 1.5× magnification: (**a**,**b**) round calcareous contact edge; (**c**–**e**) round metamorphic contact edge; (**e**) crushed calcareous aggregate contact edge; (**f**) crushed porphyry aggregate contact edge.

Figure 7 shows some images at 1.5× magnification on thin sections which represent the contact edges between the paste and the aggregate for the available lithologies. The images show that there is no interpenetration between the two elements but only a simple direct contact with a continuous and regular trend. In some cases, the cement paste does not seem to have interacted with the aggregate to such an extent as to highlight almost a total detachment between the two components. However, it must be remembered that the fragments being analyzed are obtained from the fracture of the concrete, and therefore such detachments may be consequent to the breaking of the cube. Despite this, it is possible to note that the edges are not jagged but continuous and regular, like the surface of the rounded aggregates (Figure 7a–d).

On the other hand, the fragments with crushed aggregates have a greater paste/aggregate compenetration. This apparent interrelation between the two components is seen above all on the fragments with porphyry aggregate. In fact, in Figure 7e,f, we can see how the irregular course of the aggregate surface is occupied by the cement paste, thus a sign that the mechanical adhesion is clearly better than that offered by an aggregate with a rounded surface.

From the comparison of the photo images in Figure 7, it is therefore evident that crushed aggregates respond differently to rounded aggregates in the relationship between cement paste and stone material.

## 4. Discussion

The final comparison of the data obtained made it possible to identify some mixtures of aggregates that could provide good physical-mechanical and lithological characteristics to concrete.

**Figure 8:**
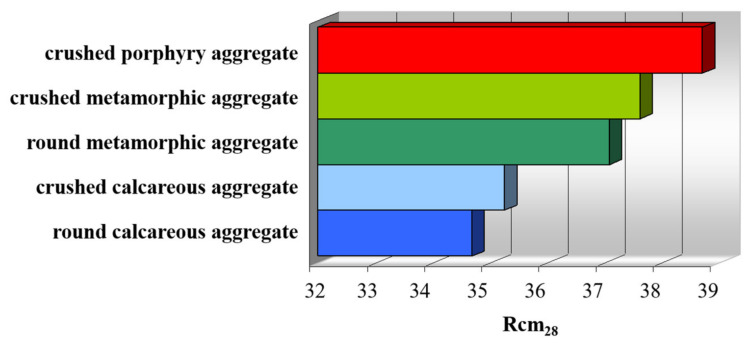
Comparison of the average values of compressive strengths (Rcm_28_) on the five different mixtures obtained: red corresponds to crushed porphyry aggregate; light green to crushed metamorphic aggregate; green to round metamorphic aggregate; light blue to crushed calcareous aggregate; blue to round calcareous aggregate.

The analysis of the average compressive strengths reported in Table 16 and represented in Figure 8 allowed us to highlight some important aspects. There is a growth trend in values passing from calcareous lithologies to metamorphic and porphyry. These values have small differences, of about 4 N/mm^2^, between calcareous and porphyry, but which are still significant since all the mixtures were prepared with the same parameters (same percentage of sand, same cement, same w/c ratio, etc.).

A possible correlation between compressive strength results and the Los Angeles values was therefore analyzed. The Los Angeles test is a mechanical test that could provide information on the intimate resistance of the aggregates subjected to crushing [73]. As can be seen in Table 11, calcareous aggregates present a lower resistance value than metamorphic and porphyry (remember that lower resistance coincides with higher LA values). However, it is a low difference but which allows a first consideration on the quality of the three lithologies compared. With the same w/c ratio and maturation conditions of the sample cubes, the compressive strengths confirm the best mechanical response in the porphyry aggregate with respect to the other two lithologies [74].

The graph in Figure 8 shows that crushed aggregates in two lithologies have resistance values Rcm_28_ higher than the corresponding rounded aggregates [75]. In this case, it is necessary to remember the macroscopic and microscopic observations of the concrete mixtures investigated (Figure 6 and Figure 7): rounded aggregates have extremely simple contact edges with the cement paste, that is, continuous and free of irregular patterns with asperities and recesses that are instead observed along the surfaces of the crushed aggregates. These edges, probably resulting from the crushing process produced by compression and from the mineralogical structure of each type of rock, contribute to increasing the surface exposed and made available to the cement paste to attach itself to the clasts. As observed from the analysis on the thin section by a stereomicroscope (Figure 7), the fractures follow an irregular trend as the size of the minerals within the types of rocks analyzed is greater [76], with conchoidal fractures and smooth in microcrystalline calcareous samples and very rough fractures in porphyry.

To support this, the VL values proposed in Table 13 can be considered. With this test, it is possible to obtain important information on the macro and micro roughness of the aggregates [77]. It depends on the internal texture of the type of rock, and this can certainly be reflected in the observed resistance values [78]. From the comparison of the rounded types, lower VL values of the respective crushed aggregates are observed, and thus it could be concluded that the abrasion produced by external agents must have produced smooth surfaces with no surface imperfections [79]. Clearly, in nature, this depends on the intensity of the external agents of the environment [80] and on the persistence of the abrasive action on the aggregates [81]. Aggregates with a rougher and irregular surface will offer greater resistance and therefore a greater VL [82]. Considering the stereomicroscope observations (Figure 7), it could be hypothesized that the increase in resistance of the crushed aggregates is due to the greater availability of specific surface made available which could create a greater interpenetration between the aggregate and cement paste able to withstand greater resistances.

Table 17 reports the main data obtained summarizing the average, maximum and minimum Rcm_28_ values for each type of mixture. In addition, the range of variability is observed, that is, the numerical interval within which all the data obtained are found, between the maximum and minimum resistance values. As can be seen, there is a greater range of variability for mixtures made with calcareous lithologies (both rounded gravels and crushed stones) and for porphyry while lower values for mixtures made with metamorphic aggregates. In any case, all the ranges of variability are restricted probably due to the fact that the samples have comparable mechanical strengths. However, it is important to consider that the presence of a range of variability is physiological and probably caused by the package of the cubes [83].

The standard deviation represents the average distance of the data from their mean value. This statistical data confirm the goodness of the average obtained. It is possible to obtain two equal averages from different concentrated data: the best data distribution is the one with the smallest standard deviation, which means that the values are not so different from their average. The standard deviation values shown in Table 17 confirm that the data obtained are concentrated around the average of the compressive strength value obtained.

Another parameter to determine the correct distribution of the data is the variation coefficient, expressed as a percentage ratio between the standard deviation and the average of the measurements. Low coefficient-of-variation values indicate that the concrete cubes were packaged with accuracy [84]. Low coefficients of variation are therefore observed for porphyry and metamorphic samples, while slightly higher for mixtures with calcareous aggregates (Table 17).

**Figure 9:**
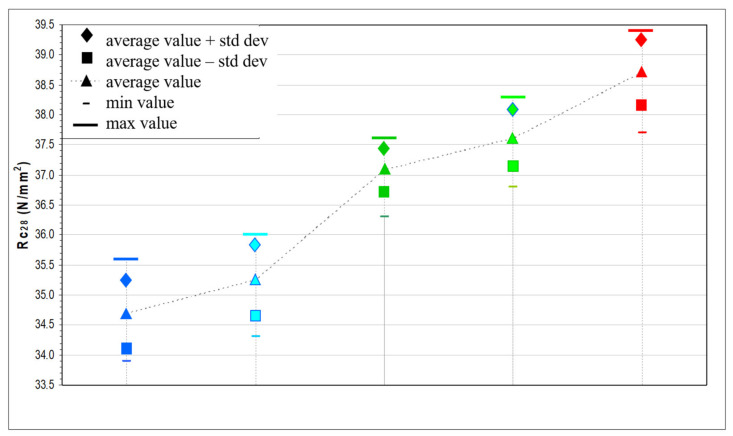
Graphical comparison of the average values plus and minus the standard deviation of the compressive strength value (Rcm_28_) of the five different mixtures obtained: red corresponds to crushed porphyry aggregate; light green to crushed metamorphic aggregate; green to round metamorphic aggregate; light blue to crushed calcareous aggregate; blue to round calcareous aggregate.

The graph in Figure 9 shows the trend of the average compressive strength values for each mixture with the respective ranges of variability. From the comparison of the intervals provided by the values of average resistance plus standard deviation and average resistance minus standard deviation, ranges of comparable amplitude are observed especially for mixtures with porphyry aggregates and with metamorphic gravels and metamorphic crushed stones, while for the calcareous aggregates, slightly wider ranges are observed.

From all this information, it can be said that the resistance data obtained are well concentrated around the representative average value for each concrete mixture and also confirm the optimal packaging of the samples [85].

## 5. Conclusions

This research work was focused on the petrographical and physical-mechanical characterization of three natural aggregates, lithologically very different from each other.

Experimental research presented included testing of petrographic properties of natural aggregates and demonstrating the relevance of their characterization in the concrete mixture.

The morphology of the aggregate clasts is important in conferring mechanical strength to the concrete, and consequently, the type and shape of the chosen lithology become a fundamental factor. This research work showed that mixtures with crushed aggregates have higher mechanical strength than mixtures with round aggregates. Unfortunately, however, the vast majority of Italian concrete mixing plants use rounded aggregates since most of the aggregate quarries are made up of alluvial sedimentary deposits characterized by gravels. In addition, the costs for crushing the extracted rounded alluvial material would be minor and not very advantageous.

The physical-mechanical data indicated good results of the porphyry aggregate with respect to the other lithologies. Furthermore, it seems that better mechanical characteristics can be obtained by the crushed aggregates thanks to their rough surface. These conclusions are valid for the w/c ratio used, for the lithologies compared and for the maturation of the concrete cubes for 28 days. Despite this, the porphyry aggregate consists of a vitreous silicate component that over time can probably degrade the durability of the concrete through chemical reactions such as alkali–silica, which are particularly harmful.

As concerns the compressive strength values of each mixture, it was observed that all the breaking values exceeded the minimum resistance limit of 30 N/mm^2^ imposed by the UNI EN 11104:2016. Furthermore, a substantial growth trend was observed, passing from calcareous to metamorphic and porphyritic lithology.

To better characterized this, further analyses are needed especially of blends packaged with different w/c ratios. It is, in fact, possible to foresee that this difference is no longer observable for high-performance concrete mixtures, with low w/c ratios and high mechanical strengths, since the cement present in considerable quantities could reduce the effects due to the petrographic composition and the form factor of the aggregates used for the mixture. In addition, it is necessary to observe the trend of the data also for maturation of the concrete cube for more than 28 days imposed by the legislation to verify that the alkali–silica reactions cannot arise with the vitreous paste of the porphyry aggregates.

Another important aspect is the necessary use of higher water contents, with the same consistency class, with a crushed aggregate compared to a rounded aggregate. This increase in water is necessary by the lower smoothness of the crushed surfaces which, being rougher, make the concrete more viscous. It is therefore deduced that crushed aggregates offer greater resistance to flow than those with rounded aggregates. This would not be a problem if there was not a consequent increase in the cement content needed to maintain the required w/c ratio, but this would mean an increase in the final cost of the mixture, largely due to the cost of this component. This would produce an uneconomical and unsaleable concrete. It should also be remembered that a concrete that is easily workable is preferred, a characteristic guaranteed by the right w/c ratio and, in addition to the additives, by the use of an alluvial aggregate with smooth surfaces. The minimum limits in the dosages of cement and w/c ratios imposed by the UNI EN standards for the packaging of a specific type of concrete, however, ensure the achievement of the mechanical resistances, and therefore it is not necessary to use aggregates with characteristics such as the surface macro roughness of the porphyry.

Ultimately it is always necessary to find the right way to relate experimental deductions deriving from studies such as the one proposed and the real practical needs imposed by the world of work, with a focus on the economic factor of the entire production process.

## Figures and Tables

**Table 1:** Aggregate mixture with calcareous gravel. On the left side, the quantity passing through the sieve expressed in percentage of the calcareous gravel and the sieve size used; on the right side, the component used for the mixture.

Sieve Size (mm)	Quantity Passing through the Sieve (%)	Mixture		
		(Quantity by Weight per 1 m^3^ of Mixture)		
31.5	100	Calcareous sand 0/1	5%	96 kg
25	100	Calcareous sand 0/6	46%	896 kg
20	87.6	Calcareous gravel 6/16	25%	489 kg
16	79.2	Calcareous gravel 16/32	24%	469 kg
14	72.5	Concrete CEM IIB LL 32.5R		300 kg
12.5	67.4	Water		173 kg
10	59.5	Fluidifying additive 0.8%		2.4 kg
8	55.2	Ratio w/c		0.58
6.3	51.9	Bulk density		2426 kg/m^3^
4	42.7	-		-
2	29.7	-		-
1	22.9	-	-	
0.5	18.5	-	-	-
0.25	10.0	-	-	-
0.125	4.6	-	-	-
0.063	2.8	-	-	-

**Table 2:** Aggregate mixture with calcareous crushed stone. On the left side, the quantity passing through the sieve expressed in percentage of the calcareous crushed stone and the sieve size used; on the right side, the component used for the mixture.

Sieve Size (mm)	Quantity Passing through the Sieve (%)	Mixture		
		(Quantity by Weight per 1 m^3^ of Mixture)		
31.5	100	Calcareous sand 0/1	5%	94 kg
25	99.1	Calcareous sand 0/6	46%	885 kg
20	88.3	Calc. crushed stone 4/8	2%	39 kg
16	77.6	Calc. crushed stone 8/12	14%	272 kg
14	73.6	Calc. crushed stone 12/20	9%	175 kg
12.5	70.8	Calc. crushed stone 20/28	24%	467 kg
10	64.2	Concrete CEM IIB LL 32.5R		310 kg
8	56.4	Water		179 kg
6.3	53.3	Fluidifying additive 0.8%		2.5 kg
4	43.2	Ratio w/c		0.58
2	30.0	Bulk density		2424 kg/m^3^
1	23.1	-		-
0.5	18.7	-		-
0.25	10.2	-	-	-
0.125	4.8	-	-	-
0.063	3.0	-	-	-

**Table 3:** Aggregate mixture with metamorphic gravel. On the left side, the quantity passing through the sieve expressed in percentage of the metamorphic gravel and the sieve size used; on the right side, the component used for the mixture.

Sieve Size (mm)	Quantity Passing through the Sieve (%)	Mixture		
		(Quantity by Weight per 1 m^3^ of Mixture)		
31.5	100	Calcareous sand 0/1	5%	96 kg
25	99.0	Calcareous sand 0/6	46%	896 kg
20	94.0	Metam. Gravel 6/16	25%	504 kg
16	84.7	Metam. Gravel 16/32	24%	480 kg
14	78.3	Concrete CEM IIB LL 32.5R		300 kg
12.5	72.6	Water		173 kg
10	63.2	Fluidifying additive 0.8%		2.4 kg
8	57.2	Ratio w/c		0.58
6.3	53.7	Bulk density		2451 kg/m^3^
4	42.9	-		-
2	30.0	-		-
1	23.2	-	-	
0.5	18.8	-	-	-
0.25	10.3	-	-	-
0.125	4.9	-		-
0.063	3.0	-	-	-

**Table 4:** Aggregate mixture with metamorphic crushed stone. On the left side, the quantity passing through the sieve expressed in percentage of the metamorphic crushed stone and the sieve size used; on the right side, the component used for the mixture.

Sieve Size (mm)	Quantity Passing through the Sieve (%)	Mixture		
		(Quantity by Weight per 1 m^3^ of Mixture)		
31.5	100	Calcareous sand 0/1	5%	94 kg
25	97.8	Calcareous sand 0/6	46%	885 kg
20	86.5	Metam. Crushed stone 5/9	5%	99 kg
16	79.4	Metam. Crushed stone 9/16	22%	434 kg
14	73.8	Metam. Crushed stone16/25	22%	438 kg
12.5	67.9	Concrete CEM IIB LL 32.5R		310 kg
10	59.6	Water		179 kg
8	56.3	Fluidifying additive 0.8%		2.5 kg
6.3	54.5	Ratio w/c		0.58
4	43.5	Bulk density		2442 kg/m^3^
2	29.9	-		-
1	23.0	-		-
0.5	18.6	-		-
0.25	10.2	-	-	-
0.125	4.7	-	-	-
0.063	2.9	-	-	-

**Table 5:** Aggregate mixture with porphyry. On the left side, the quantity passing through the sieve expressed in percentage of the porphyry and the sieve size used; on the right side, the component used for the mixture.

Sieve Size (mm)	Quantity Passing through the Sieve (%)	Mixture		
		(Quantity by Weight per 1 m^3^ of Mixture)		
31.5	100	Calcareous sand 0/1	5%	94 kg
25	99.1	Calcareous sand 0/6	46%	885 kg
20	91.6	Porphyry 4/8	7%	128 kg
16	80.0	Porphyry 8/20	30%	555 kg
14	72.3	Porphyry 16/31	12%	221 kg
12.5	67.6	Concrete CEM IIB LL 32.5R		310 kg
10	61.0	Water		179 kg
8	58.3	Fluidifying additive 0.8%		2.5 kg
6.3	56.2	Ratio w/c		0.58
4	44.0	Bulk density		2374 kg/m^3^
2	29.9	-		-
1	23.0	-		-
0.5	18.6	-		-
0.25	10.1	-	-	-
0.125	4.7	-	-	-
0.063	2.9	-	-	-

**Table 6:** Particle size dimension of calcareous gravel, calcareous crushed stone, calcareous sand and sand samples analyzed, expressed in percentage for each sieve selected and expressed in mm.

Sieve Size (mm)	Quantity Passing through the Sieve (%)							
	Calcareous Gravel 6/16	Calcareous Gravel 16/32	Calcareous Crushed Stone 4/8	Calcareous Crushed Stone 8/12	Calcareous Crushed Stone 12/20	Calcareous Crushed Stone 20/28	Calcareous Sand 0/6	Calcareous Sand 0/1
31.5	100	100	100	100	100	100	100	100
25	100	75.8	100	100	100	96.3	100	100
20	100	48.3	100	100	98.1	52.0	100	100
16	100	13.4	100	100	80.8	13.7	100	100
14	85.7	0.2	100	100	57.1	5.9	100	100
12.5	65.4	0.2	100	100	35.3	2.5	100	100
10	34.1	0.2	100	77.5	1.7	0.9	100	100
8	16.9	0.2	99.5	23.4	0.4	0.6	100	100
6.3	3.9	0.2	89.7	2.5	0.4	0.5	99.9	100
4	0.3	0.2	15.2	0.7	0.2	0.5	81.9	100
2	0.3	0.2	1.9	0.5	0.2	0.5	53.8	100
1	0.3	0.2	0.7	0.3	0.2	0.5	39.0	100
0.500	0.3	0.2	0.5	0.2	0.2	0.5	29.8	96.2
0.250	0.3	0.2	0.5	0.2	0.2	0.5	19.1	25.0
0.125	0.3	0.2	0.5	0.2	0.2	0.5	9.7	2.9
0.063	0.3	0.2	0.5	0.1	0.1	0.5	6.0	1.7

**Table 7:** Particle size dimension of metamorphic gravel, metamorphic crushed stone and porphyry samples analyzed, expressed in percentage for each sieve selected and expressed in mm.

Sieve Size (mm)	Quantity Passing through the Sieve (%)							
	Metamorphic Gravel 5/16	Metamorphic Gravel 15/30	Metamorphic Crushed Stone 5/9	Metamorphic Crushed Stone 9/16	Metamorphic Crushed Stone 16/25	Porphyry 4/8	Porphyry 8/20	Porphyry 16/30
31.5	100	100	100	100	100	100	100	100
25	100	95.8	100	100	89.8	100	100	92.2
20	100	75.0	100	100	38.8	100	97.9	35.2
16	100	36.4	100	96.2	10.2	100	72.0	3.4
14	95.6	14.0	100	79.1	2.0	100	47.4	0.9
12.5	81.9	4.7	100	53.3	0.7	100	31.8	0.4
10	47.5	1.2	100	15.8	0.4	100	9.9	0.3
8	24.3	0.6	95.6	2.2	0.2	98.1	1.6	0.3
6.3	10.4	0.5	69.4	0.3	0	73.4	0.5	0.3
4	0.7	0.4	16.6	0.2	0	16.9	0.4	0.3
2	0.6	0.4	2.8	0.2	0	0.4	0.3	0.3
1	0.6	0.4	1.0	0.2	0	0.1	0.3	0.3
0.500	0.6	0.4	0.7	0.2	0	0.1	0.3	0.3
0.250	0.6	0.4	0.6	0.2	0	0.1	0.2	0.3
0.125	0.6	0.4	0.6	0.2	0	0.1	0.1	0.2
0.063	0.5	0.3	0.3	0.1	0	0.1	0	0.1

**Table 8:** Shape index (SI) and flattening coefficient (FC) of the samples analyzed and expressed in percentage [33].

Samples	SI (%)	FC (%)
Calcareous gravel 6/16	6.4	10.5
Calcareous gravel 16/32	5.4	9.2
Calcareous crushed stone 4/8	8.2	11.7
Calcareous crushed stone 8/12	6.7	9.8
Calcareous crushed stone 12/20	5.9	8.1
Calcareous crushed stone 20/28	5.5	8.0
Metamorphic gravel 5/16	8.5	12.2
Metamorphic gravel 15/30	7.7	11.3
Metamorphic crushed stone 5/9	8.8	13.9
Metamorphic crushed stone 9/16	7.4	10.9
Metamorphic crushed stone 16/25	7.1	9.7
Porphyry 4/8	9.8	13.2
Porphyry 8/20	7.7	9.3
Porphyry 16/31	6.5	8.4

**Table 9:** ES value (expressed in percentage) [37] and methylene blue (MB) value (expressed by the ratio between g of the color and the kg of the sample) [36] for the sand samples analyzed.

Samples	ES (%)	MB (g_color_/Kg_sample_)
Calcareous sand 0/6	77.3	0.71
Calcareous sand 0/1	95.2	0.50

**Table 10:** Bulk density (ρ_a_ = apparent particle density; ρ_rd =_ particle density on an oven-dried basis; ρ_ssd_ = particle density on a saturated and surface-dried basis (expressed in Kg/m^3^)) and water absorption (WA, expressed in percentage) values determined in the analyzed samples [24].

Samples	ρ_a_ (Kg/m^3^)	ρ_rd_ (Kg/m^3^)	ρ_ssd_ (Kg/m^3^)	WA_24_ (%)
Calcareous gravel 6/16	2781	2707	2734	0.89
Calcareous gravel 16/32	2804	2692	2.732	0.98
Calcareous crushed stone 4/8	2794	2720	2747	0.98
Calcareous crushed stone 8/12	2802	2707	2749	0.80
Calcareous crushed stone 12/20	2798	2713	2753	0.73
Calcareous crushed stone 20/28	2775	2709	2757	0.69
Metamorphic gravel 5/16	2850	2795	2818	0.71
Metamorphic gravel 15/30	2833	2773	2794	0.75
Metamorphic crushed stone 5/9	2852	2782	2817	0.86
Metamorphic crushed stone 9/16	2846	2768	2795	0.63
Metamorphic crushed stone 16/25	2841	2772	2816	0.72
Porphyry 4/8	2635	2560	2592	1.27
Porphyry 8/20	2657	2594	2618	0.91
Porphyry 16/31	2645	2582	2606	0.92
Calcareous sand 0/1	2720	2649	2675	0.99
Calcareous sand 0/6	2770	2695	2722	1.01

**Table 11:** Los Angeles coefficients (LA, expressed in percentage) of the aggregates analyzed [25].

Samples	LA (%)	
	Gravel	Crushed Stone
Calcareous aggregates	19.8	20.7
Metamorphic aggregates	19.0	18.6
Porphyry aggregates	-	18.3

**Table 12:** Wear-resistance coefficient for Micro-Deval (MDE, expressed in percentage) of the aggregates analyzed [23].

Samples	MDE (%)	
	Gravel	Crushed Stone
Calcareous aggregates	6.1	8.0
Metamorphic aggregates	9.2	10.6
Porphyry aggregates	-	7.2

**Table 13:** Polishing values (PL) of the aggregates analyzed [26].

Samples	PL	
	Gravel	Crushed Stone
Calcareous aggregates	36	39
Metamorphic aggregates	45	49
Porphyry aggregates	-	53

**Table 14:** Resistance-to-freezing/thawing values (F, expressed in percentage) of the aggregates analyzed [27].

Samples	F (%)	
	Gravel	Crushed Stone
Calcareous aggregates	0.2	0.1
Metamorphic aggregates	0.1	0.1
Porphyry aggregates	-	0.2

**Table 15:** Data value of slump (expressed in mm) and bulk density (expressed in kg/m^3^) of the fresh concrete samples.

Samples	Slump (mm)	Consistency Class [47]	Bulk Density (kg/m^3^)
Calcareous gravel	189	S4	2432
Calcareous crushed stone	178	S4	2421
Metamorphic gravel	195	S4	2457
Metamorphic crushed stone	170	S4	2443
Porphyry	166	S4	2381

**Table 16:** Compressive strength values of each concrete cube after 28 days (Rcm_28_) and expressed in N/mm^2^.

Lithology	Calcareous		Metamorphic		Porphyry
Geometry	Round	Crushed	Round	Crushed	Crushed
Cube 1	34.2	35.7	37.4	37.9	38.1
Cube 2	35.4	35.4	37.0	37.6	39.4
Cube 3	34.9	34.7	37.3	37.3	39.4
Cube 4	35.1	35.0	36.9	37.9	38.5
Cube 5	35.6	35.7	37.6	37.6	38.7
Cube 6	33.9	35.9	36.3	38.3	37.7
Cube 7	34.1	35.2	37.1	37.1	39.0
Cube 8	34.2	34.3	37.4	37.4	38.7
Cube 9	34.6	36.0	36.7	38.2	38.4
Cube 10	34.8	34.5	37.1	36.8	39.2
Average Rcm_28_	34.7	35.2	37.1	37.6	38.7

**Table 17:** Comparison of the compressive strength value (Rcm_28_) of the five different mixtures analyzed, with the maximum and minimum values, variability, standard deviation (all these values expressed in N/mm^2^) and the variation coefficient expressed in percentage.

	Round Calcareous	Crushed Calcareous	Round Metamorphic	Crushed Metamorphic	Crushed Porphyry
Rcm_28_ (N/mm^2^)	34.7	35.2	37.1	37.6	38.7
Rc max (N/mm^2^)	35.6	36.0	37.6	38.3	39.4
Rc min (N/mm^2^)	33.9	34.3	36.3	36.8	37.7
Variability (N/mm^2^)	1.7	1.7	1.3	1.5	1.7
Standard deviation	0.58	0.60	0.38	0.48	0.56
Variation coefficient (%)	1.67	1.70	1.03	1.27	1.44

## Data Availability

Data sharing is not applicable.
